# Single-crystalline ZnO sheet Source-Gated Transistors

**DOI:** 10.1038/srep19232

**Published:** 2016-01-13

**Authors:** A. S. Dahiya, C. Opoku, R. A. Sporea, B. Sarvankumar, G. Poulin-Vittrant, F. Cayrel, N. Camara, D. Alquier

**Affiliations:** 1 Université François Rabelais de Tours, CNRS, GREMAN UMR 7347, 16, rue pierre et marie Curie, 37071 Tours, France; 2 Advanced Technology Institute, University of Surrey, Guildford, Surrey, GU2 7XH, United Kingdom; 3 Université François Rabelais de Tours, INSA-CVL, CNRS, GREMAN UMR 7347, 3 rue de la Chocolaterie, CS 23410, 41034 BLOIS Cedex, France

## Abstract

Due to their fabrication simplicity, fully compatible with low-cost large-area device assembly strategies, source-gated transistors (SGTs) have received significant research attention in the area of high-performance electronics over large area low-cost substrates. While usually based on either amorphous or polycrystalline silicon (α-Si and poly-Si, respectively) thin-film technologies, the present work demonstrate the assembly of SGTs based on single-crystalline ZnO sheet (ZS) with asymmetric ohmic drain and Schottky source contacts. Electrical transport studies of the fabricated devices show excellent field-effect transport behaviour with abrupt drain current saturation (I_DS_^SAT^) at low drain voltages well below 2 V, even at very large gate voltages. The performance of a ZS based SGT is compared with a similar device with ohmic source contacts. The ZS SGT is found to exhibit much higher intrinsic gain, comparable on/off ratio and low off currents in the sub-picoamp range. This approach of device assembly may form the technological basis for highly efficient low-power analog and digital electronics using ZnO and/or other semiconducting nanomaterial.

The last decade has seen resurgence in the popularity of an already matured field of nanotechnology. This is expected to aid the development of next generation efficient electronic devices incorporating nanostructures. As miniaturization *via* top down approach is nearing its limits for commercial viability, efforts are made to develop new materials with enhanced functionalities as fundamental components in future electronic/optoelectronic devices^1^. Significant developments in the synthesis of functional nanometrials *via* self-assembly from the bottom-up approach^1^,^2^ is now offering high quality materials. Additionally, the discovery and possible isolation of atomic level thick two-dimensional (2D) Graphene sheet has also spearheaded a new revolution in nanomaterial research targeting novel electronic devices and systems^3^. However, the main drawback limiting the widespread use of Graphene is the material zero band-gap^4^. This limitation of Graphene has seen a resurgence in research activities dedicated to other 2D semiconducting nanomaterials such as MoS_2_, WS_2_ and ZnO, due to their unique electrical^5^,^6^, optical^7^,^8^ and magnetic^5^,^6^ properties. While several literature data exists on the charge transport properties^8^,^9^,^10^,^11^,^12^,^13^ in such materials, the study of charge transport in single-crystalline ZnO nanosheets (NSs) is still limited^12^,^13^. This is surprising, since ZnO exhibits number of unique electrical and optical properties such as wide band-gap (3.34 eV), high exciton binding energy (60 meV), excellent thermal stability, and moderate to high electron carrier mobility (~200 cm^2^V^−1^s^−1^). Unlike other 2D semiconducting materials^14^,^15^, extraction of atomic layer ZnO is still challenging, although the growth of a few nanometer thick single-crystalline ZnO NSs^5^ have been demonstrated using bottom-up approaches. Such 2D ZnO NSs have gained significant attention for applications like: photovoltaics^16^, gas^17^/UV^18^ sensors and piezoelectric nanogenerators^19^. Since ZnO in the form of poly-crystalline thin films and single-crystalline nanowires (NWs) has been extensively studied as potential materials for the assembly of high performance field-effect transistors, targeting low-power applications^20^,^21^,^22^,^23^,^24^,^25^, it is envisaged that 2D single-crystalline ZnO NSs could offer additional functionalities in this area of nanomaterial research.

Conventionally, field-effect transistors (FETs) are engineered with ohmic source and drain (s/d) contacts. More recently, a new type of transistors operation has been introduced by Shannon and Gerstner^26^, called “source-gated transistor (SGT)”, which exploited the reverse bias Schottky diode located at the source region with markedly different saturation characteristics to that of conventional FETs with ohmic contacts^26^,^27^,^28^,^29^,^30^. A schematic of a typical SGT device is shown in Fig. 1. Of significant importance to the successful operation of SGT is the configuration of the gate electrode, which must extend across the entire portion of the semiconductor sandwiched by the Schottky source and the gate insulator layer^26^.

Exploitation of stable Schottky source/drain contacts in nanostructure based FETs^31^,^32^ (SB-FETs) have already been performed in the past. However, the device principle of SGTs is greatly different from SB-FETs even though both devices exploit a Schottky barrier at the source contact. The operating principle of the SGT can be ascribed to its geometry, which allows effective manipulation of the depleted region in the semiconductor near the vicinity of the Schottky source by the gate field (with applied gate voltage, V_GS_). In contrast to conventional FETs, the SGT exploits the reverse bias Schottky barrier at the source to afford much lower saturation voltages even at high gate voltages. This effect leads to several advantages, some of which includes: (i) early drain current saturation^26^,^28^,^33^ with the drain voltage (V_DS_), (ii) immunity of drain current (I_DS_) to channel length variations^33^, and (iii) possible immunity to short-channel effects^30^. This key defining feature of the SGT could be ideal in the design of low-power electronics, where fast switching is not necessarily a key objective.

The design of SGTs have been realized in a few semiconducting modules, namely, amorphous Si (α-Si)^28^, poly-crystalline Si (poly-Si)^27^,^34^ and poly-crystalline ZnO^22^,^23^. However, almost no data exist on the use of nanostructures or single crystals as active semiconducting modules in SGT assembly. In this work, we demonstrate fully operational SGTs based on single-crystalline ZnO sheets (ZSs), which to the best of our knowledge, has not been reported so far. Detailed discussions will be centred around the ZS growth, nanomaterial characterization and the assembly of single-crystalline ZS based SGTs. Finally, detailed electrical transport studies for the fabricated devices will be presented as follows: Schottky barrier height evaluation, field-effect transport, mobility extraction and the modes of SGT operation.

## Results

### Material structural characterizations

To assess the quality of the materials produced, we employed SEM, Raman spectroscopy, and HRTEM, as shown in shown in Fig. 2. Figure 2a shows a typical SEM image of as-grown ZS on Au-coated Sapphire substrate. The ZSs have a triangular shape and their lengths were deduced from assessment of SEM images to be around 5 μm after 60 min of synthesis. Much longer ZS on the order of 25 μm were obtained after 180 min of synthesis^13^. To determine the crystal quality and orientation of as-grown ZSs, micro-Raman and HRTEM measurements were performed. The room temperature Raman scattering was observed by a confocal microscope (×100 objective) using a 514.5 nm polarized line. The diameter of the resulting laser spot was around 1 μm, which was much smaller than the dimensions of the single ZS deposited on oxide. From this Raman data (Fig. 2b), the two dominant peaks centred at 98.6 and 437.4 cm^−1^ correspond only to investigated single ZS. These peaks are assigned to the two nonpolar first-order Raman active E_2_ (low) and E_2_ (high) modes, corresponding to the Raman selection rule of wurtzite ZnO (with C_6v_ point group symmetry). These E_2_ modes are dominating the Raman scattering spectra^35^, in accordance with a high crystal ZS material, as confirmed by the very small values of full width half maximum (FWHM) of the measured peaks: 2.2 and 5.4 cm^−1^, respectively. Finally, HRTEM characterizations were performed on single ZS to determine the ZS’s atomic structure. Figure 2c shows the low magnification HRTEM image, which was used to determine the as-grown ZS growth direction. A magnified HRTEM image of the ZS (Fig. 2c) is shown in Fig. 2(d). From this image, we were able to determine both the growth direction and other crystallographic orientation of the ZS. The data show that, the lattice fringes along the direction perpendicular to the ZS length, measured to be around 0.52 nm, corresponding to the [0001] plane of the wurtzite ZnO crystal.

Figure 3 shows a typical ZS SGT device images. Shown in Fig. 3a is a typical ZS SGT device (based on the superposition of ZS SGT schematic and AFM image). The cross-sectional TEM image of the Schottky contacted source for a representative device is shown in Fig. 3b. From this image, the Pt/n-ZS/SiO_2_/p^++^-Si stack, essential for successful SGT operation, is clearly revealed.

### Electrical characterizations

#### Pt/n-ZnO Schottky Barrier characterization at V_GS_ = 0 V

The electrical transport characteristics attained by a representative device (shown in Fig. 3a) and a schematic showing a simplified energy band diagram of the Schottky source contact region for a ZS SGT device with W/L of around 1.3/9.7 μm are shown in Fig. 4. The temperature dependent I–V measurements were performed between 303 K and 383 K in the absence of the gate field (i.e. V_GS_ = 0 V). The resulting I_DS_–V_DS_ progressions are shown in Fig. 4a. From these experimental data, it can be seen that the device demonstrates clear rectification behaviour within the measured temperature range. At RT, a rectification ratio (RR = I_DS_(−5 V)/I_DS_(+5 V)) of around 100 is calculated and a reverse saturation current I_DS_^SAT^ of ~25 nA is obtained at V_DS_ = +5 V in the present device. The Arrhenius plot for SBH extraction is shown in Fig. 4b at V_DS_ = +1.5 V. To evaluate the effective barrier height at the source Schottky Pt/n-ZnO interface, we used the pure thermionic emission (TE) model to describe the charge carrier transport; as shown in Eq. (1)


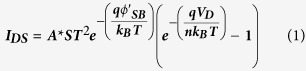


where I_DS_ is the drain-to-source current of the ZS device, A^*^ is Richardson constant corresponding to the effective mass of electrons in semiconducting ZnO, S the effective contact area, T is temperature in Kelvin, ϕ′_SB_ is the effective Schottky barrier height (SBH) and k_B_ is Boltzmann’s constant (~8.617 × 10^−5^ eV K^−1^). Using this model, the effective SBH, ϕ′_SB_ can be extracted by performing temperature-dependent I_DS_–V_DS_ measurements. Note that since A^*^ ∝ 1/ST^2^, the slope ln{(ΔI_DS_)/Δ(1/k_B_T)} from the Arrhenius plot of the current progression gives activation energy (E_a_). From this data, an activation energy was calculated from the slope of the I_DS_ progression to be ~0.28 ± 0.02 eV, which is regarded as the effective ϕ′_SB_ at the reverse biased Schottky barrier source diode. Moreover, for statistical assessment of typical SBH that can be expected in such device systems, we also extracted the effective SBH from 10 separate devices under identical experimental conditions to be in the range of 0.24 to 0.44 eV. Notably, these values are significantly lower than reported data for bulk Pt/n-ZnO Schottky contacts (0.72 eV)^36^, but still comparable to the reported data for single-crystalline ZnO nanowire Pt/n-ZnO (0.42 eV) contacts^37^. The differences between the calculated barrier height and those expected in ideal and/or bulk systems from the literature may be partly explained by our approximation of pure thermionic emission. In fact, the reverse bias current is expected to be the result of several charge transport processes at the Pt/n-ZnO interface, including: (i) recombination in depleted region of the ZS, (ii) quantum mechanical tunnel effect below the top of the barrier such as the thermionic field emission (TFE)^38^,^39^, (iii) barrier lowering due to image force effects^38^, and (iv) Fermi level pinning as a result of surface states and/or source metal-ZnO interface reactions. The combined effects of these mechanisms are expected to contribute to the injection/extraction of charge carriers at the Pt/n-ZS interface. For clarity, we show in Fig. 4c, a simplified energy band diagram of the various mechanisms resulting in the measured I_DS_ in our devices.

As a result of the successful SBH extraction, numerical simulations for our SGTs structures were performed using Silvaco Atlas, according to ref. 25. We use material parameters for unintentionally doped ZnO thin film^40^. For this investigation, a nominal Schottky barrier height of Φ_SB_ zero = 0.28 eV was assumed and the barrier lowering parameters α and γ were taken to be ~4 nm and 0.87, respectively (where SBH Φ_SB_ effective = Φ_SB_ zero–αE_γ_)^28^. The results from these simulations are discussed in the following part of the present work.

#### General field-effect characteristics: threshold voltage, leakage current, sub-threshold swing, current on/off ratio, mobility

The general field-effect characteristics exhibited by both devices: (i) a ‘standard’ FET with ohmic contacts and (ii) a SGT with Schottky source contact will now be discussed to highlight their main differences. It is worth to note that, in order to avoid any effect from different field by applying same V_DS_ in the device channel, we measured the same device with asymmetric ohmic drain and Schottky source contacts by appropriate biasing for both FET (drain grounded) and SGT (source grounded). In SGT theory, the gate field serves two purposes. Firstly, it modulates the channel conductivity such that a conduction path exists at V_GS_ ≥ V_TH_ for charge transport, as in conventional FET. Secondly, it modulates the SBH by penetrating into the semiconductor region sandwiched by the source and gate electrode^26^,^27^,^28^. As we show in subsequent sections of this work, this key defining differences leads to markedly different transport in such devices.

Experimental and numerical transfer I_DS_-V_GS_ scans at constant drain bias (V_DS_ = +1 V) for V_GS_ bias range of −25 V to 25 V are shown in Fig. 5a,b for both device types. In both cases, the general progression of I_DS_, which increases with V_GS_ bias, is typical of n-channel accumulation mode behaviour. A turn-on voltage of ~−6 V and ~−22 V is obtained for the SGT and FET devices, respectively. A linear extrapolation of I_DS_-V_GS_ (not shown) revealed threshold voltages (V_TH_) of ~−3.4 V (SGT) and ~−12 V (FET). From the semi-log plot of the transfer scans, comparable subthreshold swing ≈ [∆logI_DS_/∆V_GS_] of ~750 mV/dec was obtained in the two device types. From the logarithmic ratio of the on-to-off currents (log_10_ [I_on_/I_off_]), an on/off ratio of ~10^5^ (SGT) and ~10^7^ (FET) were obtained. The comparatively higher on/off value in the FET is expected due to the absence of a reverse bias Schottky barrier in this device. Notably, both devices demonstrate exceptionally low off state currents in the sub-picoamp (~0.1 pA). Such low currents may be attributed to the use of ohmic drain contact^22^ and/or the low free charge carrier density in the ZS used. The field-effect mobility in both devices were evaluated from the standard MOSFET model in the linear regime, as shown in Eq. 2.^41^,


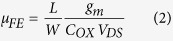


where L is the channel length, W the channel width, g_m_ the transconductance = ∂I_DS_/∂V_GS_ and C_OX_ the gate capacitance (=ε_0_ε_r_/d). From Eq. 2, we obtained a conservative estimation for the field-effect mobility (μ_FE_) ~5 cm^2^/Vs (SGT) and ~50 cm^2^/Vs (FET) for the devices. It should be noted that the extracted μ_FE_ can be regarded as an effective mobility (μ_eff_) as it does not reflect the true mobility of a contact controlled device such as the present SGT. As such, the mobility of 5 cm^2^/Vs is provided purely for comparison. Nonetheless, μ_eff_ value in the present ZS SGTs is still higher than that in reported data for other SGT device structures based on poly-ZnO^22^,^23^. Another important feature to note from the general progression of the I_DS_ (transfer scans, Fig. 5b), is their markedly different shapes. The SGT shows abrupt current saturation behaviour while for the FET shows much weaker saturation. These differences in operation can be seen much more clearly in the family of output scans for the two devices (Fig. 5(c,e), in which the SGT shows abrupt I_DS_^SAT^ of less than 2 V (V_DS_) even at high V_GS_ biases. The FET, on the other hand, shows very weak I_DS_^SAT^ behaviour. Such differences in the two device types can be directly attributed to the existence of the reverse bias Schottky barrier in the SGT which essentially controls charge carrier injection from contact-to-channel, irrespective of channel conductance (at V_GS_ ≥ V_TH_ and V_DS_ ≥ V_DS_^SAT^).

#### Abrupt current voltage saturation/Intrinsic Gain

**Figure 1:**
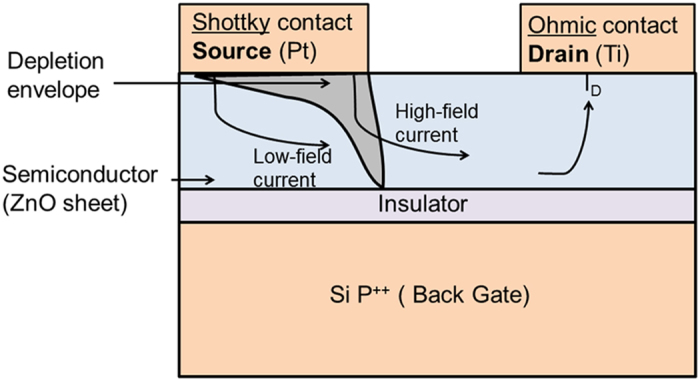
Cross-sectional device structure of a SGT. In blue are the materials used in our device. In grey is the depletion envelope at the source under small drain bias showing pinch-off. Current is controlled by the reverse biased Schottky source contact. The total current of the device itself is a contribution of two components, namely, high-field and low-field mode of operation. The high-field mode is through the modulation of barrier height present at the edge of the source while the low-field mode is through the depletion region present under bulk of the source.

**Figure 2:**
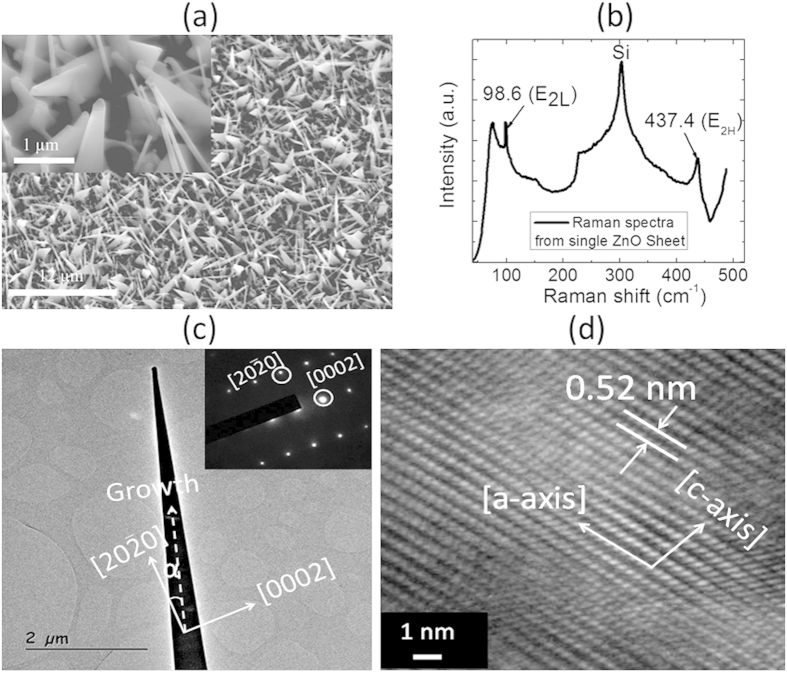
(**a**) SEM image of the as-grown ZSs on Sapphire substrate. The inset is high-magnification image of the respective sample. (**b**) Raman spectra measured from single ZS. (**c**) HRTEM image of single ZS showing the growth direction. The growth direction is always inclined with angle alpha to the a-axis. The Inset selected area electron diffraction (SAED) pattern further confirms the single-crystalline nature of the ZS. (**d**) HRTEM image of ZS.

**Figure 3:**
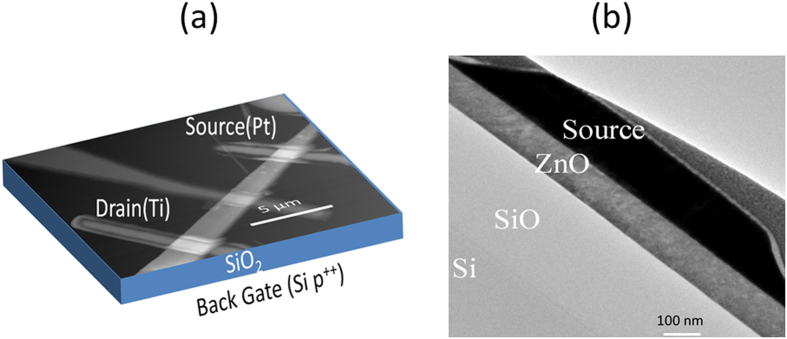
Schematic/AFM image of the ZS SGT device (b) Cross-sectional TEM image of the fabricated SGT device on 290 nm thick SiO_2_ showing the SGT’s staggered structure and ZS’s approximate thickness of 120 nm.

**Figure 4:**
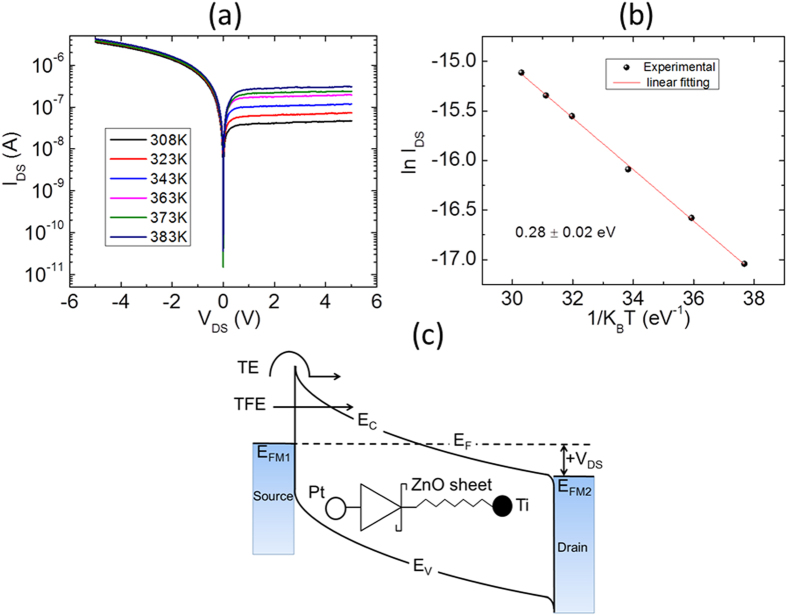
(**a**) I–V characteristics of Pt-ZnO contact as a function of temperature from 308 to 383 K. (**b**) Arrhenius plot. The activation barrier energy is extracted from the plot by measuring the slope of the curve which comes to be 0.28 ± 0.02 eV. (**c**) Simplified energy band diagram for Pt-ZnO-Ti system under thermal equilibrium at positive V_DS_. Also schematically showing the two dominant charge carrier injection mechanism (TE and TFE) at reverse biased Schottky contact. E_FM1_ and E_FM2_ are the Fermi level for metals Pt and Ti, respectively.

**Figure 5:**
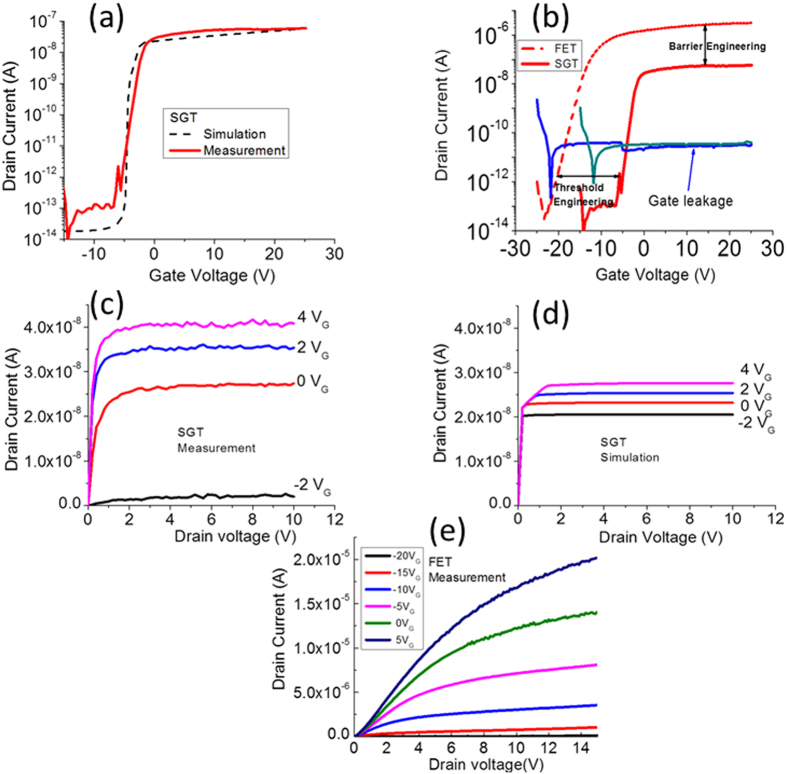
(**a**) Experimental and simulated I_DS_-V_GS_ curve of the SGT device at V_DS_ = 1 V. (**b**) I_DS_-V_GS_ curves from the Pt-ZnO (SGT) and Ti-ZnO (FET) curves of the same device at V_DS_ = 1 V. Panel (**b**) also show the gate leakage current for both devices. (**c**) Experimental and (**d**) simulated output characteristics for the SGT device with V_GS_ steps of 2 V between −2 to 4 V. (**e**) Output characteristics for the FET device with V_GS_ steps of 5 V between −20 to 5 V.

The measured and simulated output characteristics (I_DS_-V_DS_) for the present SGT and FET are depicted in Fig. 5c–e. Notably, the output scans demonstrate excellent modulation with increasing steps of V_GS_ from −2 V to + 4 V. Consistent with the progression of the transfer scans, the SGT output scans also demonstrate abrupt current saturation characteristics with increasing V_GS_. Beyond V_DS_ = V_DS_^SAT^, I_DS_ is clearly shown to remain fairly stable, invariant with increasing V_DS_ beyond saturation. Compared to the FET device (Fig. 5(e)), drain current saturation in the SGT device appears at significantly lower drain voltages, even at relatively high gate voltages. In conventional FETs with ohmic contacts, drain current saturation is expected to occur first at the drain end of the channel. This is described by the gradual channel approximation model: V_DS_^SAT^ ≈ [V_GS_–V_TH_]. According to the theory, the ratio of the change in the saturation voltage with gate voltage would be close to unity (∂V_DS_^SAT^/∂V_GS_ ≈ 1) for FETs. This is completely different for the SGT device where a ∂V_DS_^SAT^/∂V_GS_ ≈ 0.1 was obtained. This is a direct consequence of the small positive drain bias that leads to strong pinch-off near the vicinity of the reverse bias source Schottky contact (see Fig. 1). Beyond this pinch-off point, the supply of charge carriers from source to drain is all but dominated by quantum mechanical tunnelling through the reverse biased Schottky barrier and effective manipulation of the SBH by the gate field acting on it. Moreover, as can be seen from the output scan in Fig. 5c, the progression of I_DS_^SAT^ demonstrates a near-linear relationship with incremental increase of V_GS_ from V_GS_ = 0 V up to V_GS_ = 4 V. Since the physical length of the channel in the SGTs is not expected to play a significant role in charge transport at V_GS_ ≥ V_TH_ and V_DS_ ≥ V_DS_^SAT^ 27, we can conclude that SBH lowering is the primary mechanism which controls the increase of I_DS_ with increasing V_GS_. In the following sections, we also show that the barrier height become less sensitive to the gate field beyond a certain V_GS_ value (typically » V_TH_), as in the case of SGT operation in the low field regime^30^.

The features of our device with Schottky source contacts strongly resembles that of a classical SGT^26^, which was shown to offer enhanced performance of transistors targeting logic circuits as well as simple pixel switches in actively addressed liquid-crystal displays. A crucial parameter for most transistor applications is the intrinsic gain A_v_, defined as A_v_ = g_m_/g_d_ (where g_d_ = ∂I_DS_/∂V_DS_)^42^. The value of A_v_ is related to the output impedance^42^. Generally, in conventional FETs with ohmic contacts, high A_v_ values are difficult to achieve at low drain voltages unless sufficient V_DS_ bias (»V_DS_^SAT^) is applied at the drain for drain pinch-off. As shown for the SGTs, much higher A_v_ is anticipated, as I_DS_^SAT^ occurs first at the source end of the device at significantly lower V_DS_ biases. This leads to an A_v_ higher than 100 at V_DS_ = 2 V, approximately 1000 times higher than in our FET devices. It must be noted that such an abrupt current-voltage saturation and high intrinsic gain characteristics exhibited by our SGTs is expected to be useful in applications where high output impedance, good current uniformity and stability are required, such as in driver transistors in emissive pixel circuits. This is mainly because such devices generally operate in saturation to provide constant currents with some tolerance in supply voltage variations^29^.

## Discussion

**Figure 6:**
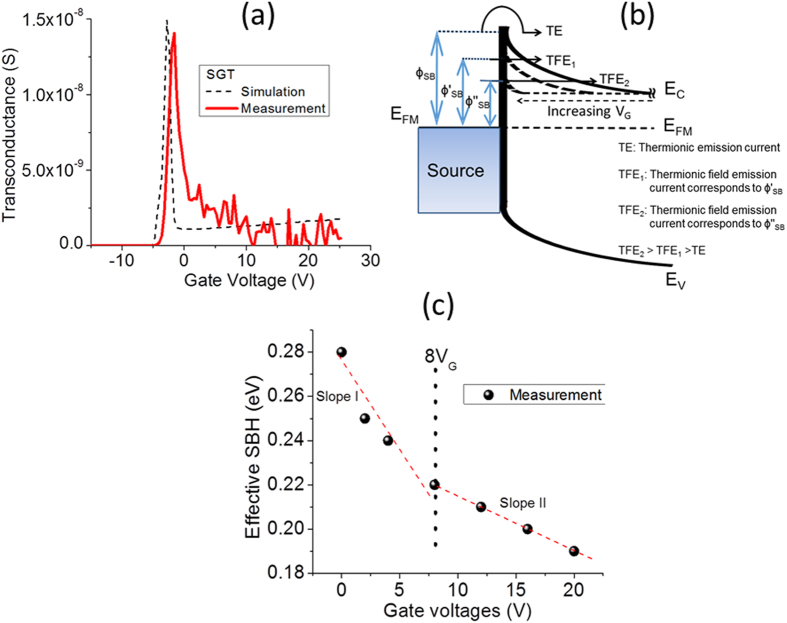
(**a**) Experimental and simulated transconductance curve of the SGT device at V_DS_ = 1 V. (**b**) Simplified energy band diagram showing variation in effective barrier height with change in applied V_GS_. (**c**) Extracted effective SBH with gate voltage variation when V_DS_ = 2 V. It can be seen that the effective SBH decreases very sharply with rate 0.007 eV/V_GS_ till V_GS_ equal to 8 V (slope I). As shown in (**c**), beyond 8 V_GS_, SBH demonstrates exceptionally low decrease rate 0.0024 eV/V_GS_ (slope II), which is approximately three times lower than SBH decrease rate below 8V_GS_.

The specific mechanism leading to charge carrier transport from contact-to-channel in our ZS SGTs can be analysed in detail by assessing the progression of the transconductance g_m_ vs. V_GS_, as shown in Fig. 6a. The general trend of g_m_ shows a maximum value of 14 nS at ~V_GS_ = −1.5 V, which then falls abruptly to around 3 nS at V_GS_ = 1 V. Similar to reported data from the literature for SGTs, the behaviour of g_m_ can be attributed to the two dominant charge carrier transport processes (TE and TFE)^39^ at the metal-semiconductor junction (Pt/n-ZS in the present case). The process of charge carrier injection/extraction at the interface can be well described by the gate potential variation at the ZS-SiO_2_ dielectric interface. In the case of a device with large global back gate, the gate potential variation can be expressed by^39^,^43^:


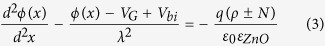



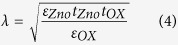


where Φ(x) represents the potential variation at the ZS channel-SiO_2_ dielectric interface, V_bi_ is the built-in potential in the depletion region, directly adjacent to the Pt/n-ZnO interface, ρ is the density of mobile carriers, N is constant charge due to ionized donors/acceptors; t_ZnO_ and t_OX_ are the thicknesses of ZS and SiO_2_, respectively, λ on the other hand can be regarded as the screening length and/or a scaling parameter. In SGTs, increasing the gate field effectively modulates the width of the depletion region in the semiconductor, provided that: V_GS_ ≥ V_TH_ and V_DS_ ≥ V_DS_^SAT^. Increasing gate field modifies the Schottky barrier in such a way that charge carrier injection at the reverse bias Schottky diode is dominated by TFE. However, note in the experimental data (Fig. 6a) that g_m_ in the present SGT demonstrates negligible increase beyond a local maxima (14 nS at V_GS_ = −1.5 V). This behaviour of the device presumably suggests that the gate no longer act strongly on the reverse bias source Schottky barrier. In fact, this contradicts the majority of reported data on SGT operation^26^,^29^,^34^, whereby the g_m_ is expected to increase slowly beyond the local maxima. According to Shannon *et al.*^30^, this can be related to a different operating regime of the SGT. In fact, computational simulation performed by the authors have revealed that the SGT can operate under two distinctive regimes that can be characterized by “high-field and low-field” modes (see Fig. 1).

The high-field operation has been suggested as being the more classical operating mode of the device, in which a greater proportion of the charge (from source to drain) originate from the edge of the source (high-field region). Under this regime g_m_ is expected to increase gradually with increasing V_GS_ even after the observed local maxima. In general, the SBH lowering is exponentially proportional to gate-field. The high-field mode can be explained using the schematic shown in Fig. 6b. From this Figure, at V_GS_ > V_TH_, the semiconductor channel is accumulated by charge carriers thus making it more conductive than the reverse bias Schottky diode, provided that sufficient V_DS_ is applied at the drain (with respect to a grounded source) to ensures that pinch-off occurs first at the source end of the device. Under such biasing conditions, the supply of charge carriers from source in to channel is expected to be dominated by a combination of TFE and image force barrier lowering (at V_GS_ > V_TH_). This regime can be regarded as the high-field and thus, the total source current is expected to be mainly due to the injection of charge carriers at the edge of the source contact. This effect gives an exponential increase of I_DS_ in the transfer characteristics^30^. However, under low-field mode, a greater proportion of I_DS_ originates from the injection of charge beyond the edge of the source (low-field region). Due to this, modulation of the SBH by the gate field is negligible. This mechanism may in part explain the low field dependence of the SBH at higher V_GS_ values from the experimental data. In low-field mode, the source current at low V_GS_ is expected to steadily increase with V_GS_ up to a local maximum. However, beyond this local maximum, the source is no longer able to supply sufficient carriers to the channel, resulting in complete I_DS_ saturation. This is in fact what can be observed in the experimental data shown in Fig. 5a, where I_DS_ shows negligible increase at V_GS_ > ∼8 V. The conditions proposed by Shannon *et al.*^30^ for low-field regime include the presence of low SBH and large source-semiconductor overlaps. The source length (2 μm) and the calculated SBH in the present SGT device (0.28 eV) satisfies the proposed SGT conditions^29^,^30^ in the present work. In an attempt to elucidate the gate field-dependent SBH lowering hypothesis, we carried out temperature-dependent output measurements of our device at gate bias voltages ranging from 0 to 20 V. The results from the temperature work are shown in Fig. 6c. From this experimental data, it can be seen that the effective SBH demonstrates exceptionally low decrease with increasing V_GS_ beyond 8 V. Based on experimental observations from Fig. 6c and the general progression of g_m_ (Fig. 6a), it is reasonable to conclude that the present device operates in the low-field SGT mode. It has been shown, both by experimental and simulation work^44^,^45^, a device operating under low-field regime has advantage of having lower activation energy (low temperature dependence) while maintaining the obvious advantages of SGTs such as low saturation voltage and high output impedance in saturation.

## Methods

### ZnO sheet growth procedure

High density of single-crystalline ZSs were obtained using a catalytic-assisted vapour-liquid-solid (VLS) process in a conventional tube furnace on a Au-coated Sapphire substrates at 950 °C^13^. To grow the ZSs, the source material (ZnO and C at 1:1 weight ratio) was first placed in an Alumina boat, which was subsequently inserted close to the centre of the quartz tube furnace. An Ar ambient was maintained inside the growth chamber throughout the whole process. To initiate the growth, the furnace was ramped to 950 °C at a ramp rate of 30 °C min^−1^, while the growth time at the plateau (950 °C) was varied from 60 to 180 min. After the growth, the furnace was switched off and left to cool naturally to room temperature and growth substrates were recovered thereafter.

### Morphological and structural characterizations

Morphological and structural characterizations of the as-grown ZSs have been performed in three different equipments. First, a dual beam FEI Strata 400 (FEI, Hillsboro, OR, USA), a focused ion beam (FIB) coupled to a scanning electron microscopy (SEM) system, has been used. It is equipped with a flip stage, a scanning transmission electron microscopy (STEM) detector, and an energy-dispersive x-ray spectroscopy for sample transfer, observation, and elemental composition characterization, accordingly. Furthermore, ZS FET device lamellas have been prepared using the FIB mode and then characterized in STEM mode, but also in second equipment: a high-resolution transmission electron microscopy (HRTEM) using a JEOL 2100 F (JEOL Ltd., Akishima-shi, Japan) operating at an accelerating voltage of 200 kV is performed.

### ZS based transistor fabrication

To fabricate the ZS SGT/FET devices, the as-grown ZSs were dispersed onto highly doped p^++^-Si substrate with 170 and/or 290 nm thick thermally grown SiO_2_ layer. Using electron-beam lithography, metallic source and drain contacts were defined on to opposite ends of a selected ZS using a two-step lithography process. In the first step, high work function metal (Pt; W = 6.1 eV) was defined as the source contact. Accordingly, the drain contact was employed with a low work function metal (Ti; W = 4.33 eV), in the step. For the present investigation, several devices were fabricated with various channel lengths (L) ranging from 1 to 10 μm. All electrical assessment of the fabricated ZS SGTs/FET were carried using a Cascade Microtech Summit 11k probe station with single source measure unit (2636A by Keithley Instruments) under dark ambient conditions.

## Conclusion

To conclude, the present work has successfully demonstrated the fabrication and electrical characterization of high performance Pt/n-ZnO SGT devices based on single-crystalline ZnO sheets. The Pt/n-ZnO diode demonstrated low reverse leakage current of around 25 nA, with a rectification ratio greater than 100 at V_DS_ = +5 V. Assessment of the field-effect transport characteristics of the fabricated SGT device revealed exceptionally low saturation voltages and 1000 times higher gain (at V_DS_ = 2 V) compared to an identical ZS FET devices. The investigated SGT device is expected to be useful in applications where high output impedance, good current uniformity and stability are required, such as in driver transistors in emissive pixel circuits. We envisage that the present ZS SGT device may offer practical solutions to realise high performance low-power electronic device based on ZnO sheets.

## Additional Information

**How to cite this article**: Dahiya, A. S. *et al.* Single-crystalline ZnO sheet Source-Gated Transistors. *Sci. Rep.* **6**, 19232; doi: 10.1038/srep19232 (2016).
